# Examining inequities associated with incarceration among breast cancer patients

**DOI:** 10.1002/cam4.7428

**Published:** 2024-08-08

**Authors:** Yoshiko Iwai, Alice Yunzi L. Yu, Samantha M. Thomas, Tyler Jones, Kelly E. Westbrook, Andrea K. Knittel, Oluwadamilola M. Fayanju

**Affiliations:** ^1^ The University of North Carolina School of Medicine Chapel Hill North Carolina USA; ^2^ Department of Pediatrics Northwestern University, Feinberg School of Medicine Chicago Illinois USA; ^3^ Department of Biostatistics and Bioinformatics Duke University School of Medicine Durham North Carolina USA; ^4^ Duke Cancer Institute Duke University School of Medicine Durham North Carolina USA; ^5^ Department of Medicine Duke University School of Medicine Durham North Carolina USA; ^6^ Department of Obstetrics and Gynecology University of North Carolina School of Medicine Chapel Hill North Carolina USA; ^7^ Department of Surgery, Perelman School of Medicine University of Pennsylvania Philadelphia Pennsylvania USA; ^8^ Rena Rowan Breast Center Abramson Cancer Center, Penn Medicine Philadelphia Pennsylvania USA; ^9^ Penn Center for Cancer Care Innovation University of Pennsylvania Philadelphia Pennsylvania USA; ^10^ Leonard Davis Institute of Health Economics University of Pennsylvania Philadelphia Pennsylvania USA

## Abstract

**Introduction:**

Breast cancer treatment patterns and quality of care among patients experiencing incarceration are underexplored. This study examined associations between incarceration and breast cancer disease and treatment characteristics.

**Methods:**

This retrospective analysis was conducted at a tertiary center in the Southeastern United States that serves as the state's safety‐net hospital and primary referral site for the state's prisons. All patients ≥18 years diagnosed with breast cancer between 4/14/2014–12/30/2020 were included. Incarceration status was determined through electronic health record review. Linear regression was used to estimate the association of incarceration with time to treatment. Unadjusted overall survival (OS) was estimated using the Kaplan–Meier method with log‐rank tests to compare groups.

**Results:**

Of the 4329 patients included, 30 (0.7%) were incarcerated at the time of diagnosis or treatment (DI) and 4299 (99.3%) had no incarceration history (NI). Compared to patients who were NI, patients who were DI were younger (*p* < 0.001), more likely to be unmarried (*p* < 0.001), and more likely to have family history of breast cancer (*p* = 0.02). Patients who were DI had an increased time from diagnosis to neoadjuvant chemotherapy (+47.2 days on average, 95% CI 3.9–90.5, *p* = 0.03) and from diagnosis to surgery (+20 days on average, 95% CI 6.5–33.5, *p* = 0.02) compared to NI patients. No difference in OS was observed (log‐rank *p* = 0.70).

**Conclusions:**

Patients who are incarcerated experienced significant delays in breast cancer care. While no differences in mortality were appreciated, these findings are concerning, as they indicate poorer care coordination for patients who are incarcerated. Further research is necessary to understand the full scope of these disparities and elucidate factors that contribute to them.

## Graphical abstract

In this retrospective cohort review at a single tertiary center in the United States and primary referral site for the state's prisons, patients who were incarcerated at diagnosis, or treatment were more likely to have a family history of breast cancer but had an average 47.2‐day increased time from diagnosis to neoadjuvant chemotherapy and 20‐day increased time from diagnosis to surgery compared to those who have never been incarcerated. Despite risk factors for breast cancer, patients who are incarcerated experience significant delays in care.

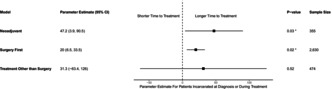

## 1 INTRODUCTION

With nearly 2 million people incarcerated in the criminal legal system, the United States (US) has one of the largest carceral populations in the world.^1^ In 2021, the Department of Justice reported cancer as the leading cause of illness‐related death in state prisons, surpassing heart disease.^2^, ^3^ A review by Aziz et al from the same year reported an incidence ratio of cancer among the confined population as two‐fold higher compared to those who were not incarcerated, after adjusting for age, sex, race, and year of diagnosis.^3^ Further, the risk of all‐cause mortality and cancer‐related death at 5 years was higher for people during incarceration and after release compared to people who had never been incarcerated.^4^ While the prevalence and incidence of cancer among incarcerated populations vary across studies,^3^, ^5^, ^6^, ^7^ the literature consistently suggests higher rates of cancer mortality among individuals who are incarcerated.^4^, ^8^

While healthcare is a constitutional right during incarceration,^9^ the size and increasing age of the US carceral population raises serious concerns for the criminal legal system's capacity to ensure adequate healthcare, and more specifically, cancer care. Several barriers have been raised as contributing to the higher rates and poorer outcomes of cancer in US jails and prisons. In most US prisons, specialty care (including cancer operations, systemic therapy, and radiation therapy) that cannot be provided on prison grounds are sourced from tertiary‐care facilities or other large‐scale medical centers. These off‐site services constitute a sizeable portion of prison healthcare expenditure.^10^ Transportation, care coordination, and financial barriers are likely contributing to patients' presenting at more advanced stages of cancer and having more interrupted, uncoordinated care when incarcerated.^3^, ^11^ Additionally, limited cancer screening in prisons and jails, underlying comorbidities, and poorer general health are likely exacerbating cancer disparities at the population level.^12^, ^13^, ^14^

Treatment patterns and quality of cancer care among prison populations remain largely underexplored.^8^ For breast cancer, studies report anywhere between 0% and 58% of women who are incarcerated have up‐to‐date mammograms,^14^, ^15^, ^16^ and the impact of inadequate screening on clinical outcomes is largely unknown given breast cancer's female predominance and that women constitute a minority of the US incarcerated population. In this study, we sought to identify and examine associations between incarceration and breast cancer disease and treatment characteristics. As individuals from racial and ethnic minority groups in the US are overrepresented in the incarcerated population^17^ and experience breast cancer disparities outside of carceral settings,^18^, ^19^, ^20^, ^21^ we hypothesized that individuals who are incarcerated would present at later stages of disease, experience delays in care, and have concomitantly worse breast cancer outcomes.

## 2 METHODS

This study was reviewed by institutional review boards at the University of North Carolina (NC) (#20–2471) and Duke University (#Pro00106936) and operated under a waiver of informed consent. The study followed the *Strengthening the Reporting of Observational Studies in Epidemiology* (STROBE) guidelines.^22^

In this retrospective analysis, patients ≥18 years old who received a breast cancer diagnosis and/or treatment at a single tertiary care center in NC were included. This tertiary care center is a safety‐net hospital and acts as the primary referral site for the state's prisons according to the Department of Adult Corrections (formerly the Department of Public Safety). The analytic cohort consisted of all patients who were diagnosed with or treated for breast cancer between 4/14/2014 and 12/30/2020. Data were obtained through the NC Translational and Clinical Sciences Institute (NC TraCS), a university‐affiliated research support service. Data custodians at NC TraCS assisted the research team with obtaining and merging electronic health record (EHR) data from the tertiary care center and state cancer registry data to abstract demographic, clinical, and treatment characteristics.

Incarceration status was determined through review of patient data in the EHR sorted by payor or insurance status (e.g., “Department of Public Safety”) and/or patient type (e.g., “prisoner”). Timing of incarceration in relation to breast cancer diagnosis and treatment were corroborated through direct EHR review. Patients were grouped as not incarcerated (NI), incarcerated before diagnosis only (BI), and incarcerated at diagnosis or during treatment (DI).

Patient demographics including incarceration status, disease characteristics, and treatment details were summarized with N (%) for categorical variables and median (interquartile range [IQR]) for continuous variables. Differences between groups were tested using the chi‐square or Fisher's exact test for categorical variables and Wilcoxon rank sum test for continuous variables. Linear regression was used to estimate the association of incarceration status with time to treatment, modeled separately by treatment sequence (neoadjuvant or upfront surgery). Unadjusted overall survival (OS) was estimated using the Kaplan–Meier method, and the log‐rank test was used to compare groups. No adjustments were made for multiple comparisons. Missingness for each variable is summarized in Table 1. All tests were conducted using complete case analysis.

**TABLE 1:** Characteristics of patients incarcerated at diagnosis or during treatment vs. not incarcerated.

Variable	Level	No. (%)^a^	Overall *N* = 4329	Incarcerated at diagnosis or during treatment *N* = 30	Not incarcerated *N* = 4299	*p*‐Value
Age at Diagnosis	Median (IQR)	4328 (100%)	60.35 (50.01, 69.35)	49.39 (41.11, 57.69)	60.44 (50.15, 69.39)	**<0.001**
Race	Black or African American	4049 (93.5%)	870 (21.49%)	9 (31.03%)	861 (21.42%)	0.41
	Other race		258 (6.37%)	1 (3.45%)	257 (6.39%)	
	White or Caucasian		2921 (72.14%)	19 (65.52%)	2902 (72.19%)	
Marital status	Divorced or legally separated	3970 (91.7%)	529 (13.32%)	2 (10.53%)	527 (13.34%)	**<0.001**
	Married or domestic partner		2194 (55.26%)	3 (15.79%)	2191 (55.45%)	
	Single		733 (18.46%)	13 (68.42%)	720 (18.22%)	
	Widowed		514 (12.95%)	1 (5.26%)	513 (12.98%)	
BMI	Median (IQR)	3096 (71.5%)	28.2 (24.12, 33.6)	28.8 (25.9, 35.7)	28.2 (24.1, 33.52)	0.30
Noted alcohol use at diagnosis	No	2801 (64.7%)	1519 (54.23%)	12 (85.71%)	1507 (54.07%)	**0.02**
	Yes		1282 (45.77%)	2 (14.29%)	1280 (45.93%)	
Noted tobacco use at diagnosis	No	3151 (72.8%)	2754 (87.40%)	20 (86.96%)	2734 (87.40%)	>0.99
	Yes		397 (12.60%)	3 (13.04%)	394 (12.60%)	
Noted substance use at diagnosis	No	2667 (61.6%)	2582 (96.81%)	12 (80.00%)	2570 (96.91%)	**0.01**
	Yes		85 (3.19%)	3 (20.00%)	82 (3.09%)	
BRCA mutation	No	4329 (100%)	4286 (99.01%)	30 (100.00%)	4256 (99.00%)	>0.99
	Yes		43 (0.99%)	0 (0.00%)	43 (1.00%)	
Family history of breast cancer	No	4329 (100%)	2525 (58.33%)	11 (36.67%)	2514 (58.48%)	**0.02**
	Yes		1804 (41.67%)	19 (63.33%)	1785 (41.52%)	
Family history of other cancers	No	4329 (100%)	1630 (37.65%)	17 (56.67%)	1613 (37.52%)	**0.03**
	Yes		2699 (62.35%)	13 (43.33%)	2686 (62.48%)	
Grade	1	2343 (54.1%)	433 (18.48%)	3 (18.75%)	430 (18.48%)	0.24
	2		1021 (43.58%)	10 (62.50%)	1011 (43.45%)	
	3		889 (37.94%)	3 (18.75%)	886 (38.07%)	
ER status	Negative	1676 (38.7%)	332 (19.81%)	3 (37.50%)	329 (19.72%)	0.20
	Positive		1344 (80.19%)	5 (62.50%)	1339 (80.28%)	
PR status	Negative	1671 (38.6%)	489 (29.26%)	3 (37.50%)	486 (29.22%)	0.70
	Positive		1182 (70.74%)	5 (62.50%)	1177 (70.78%)	
HER2 status	Negative	1450 (33.5%)	1223 (84.34%)	7 (87.50%)	1216 (84.33%)	>0.99
	Positive		227 (15.66%)	1 (12.50%)	226 (15.67%)	
Pathological stage	0	1966 (45.4%)	362 (18.41%)	5 (29.41%)	357 (18.32%)	0.42
	1		824 (41.91%)	8 (47.06%)	816 (41.87%)	
	2		588 (29.91%)	4 (23.53%)	584 (29.96%)	
	3		192 (9.77%)	0 (0.00%)	192 (9.85%)	
Clinical stage	0	1495 (34.5%)	235 (15.72%)	0 (0.00%)	235 (15.79%)	0.26
	1		812 (54.31%)	4 (57.14%)	808 (54.30%)	
	2		306 (20.47%)	1 (14.29%)	305 (20.50%)	
	3		142 (9.50%)	2 (28.57%)	140 (9.41%)	
Summary stage	0	3798 (87.7%)	573 (15.09%)	6 (22.22%)	567 (15.04%)	0.74
	1		1720 (45.29%)	12 (44.44%)	1708 (45.29%)	
	2		1070 (28.17%)	7 (25.93%)	1063 (28.19%)	
	3		435 (11.45%)	2 (7.41%)	433 (11.48%)	
Treatment Group	Neoadjuvant	3611 (83.4%)	355 (9.83%)	1 (3.85%)	354 (9.87%)	0.25
	No treatment		152 (4.21%)	3 (11.54%)	149 (4.16%)	
	Surgery first		2630 (72.83%)	19 (73.08%)	2611 (72.83%)	
	Treatment other than surgery		474 (13.13%)	3 (11.54%)	471 (13.14%)	
Breast surgery	Lumpectomy	3613 (83.5%)	1294 (35.82%)	10 (38.46%)	1284 (35.80%)	0.62
	Mastectomy		1693 (46.86%)	10 (38.46%)	1683 (46.92%)	
	None		626 (17.33%)	6 (23.08%)	620 (17.28%)	
Treatment with chemotherapy	No	4329 (100%)	2553 (58.97%)	20 (66.67%)	2533 (58.92%)	0.39
	Yes		1776 (41.03%)	10 (33.33%)	1766 (41.08%)	
Chemotherapy type	Adjuvant	1163 (26.9%)	906 (77.90%)	5 (83.33%)	901 (77.87%)	1.00
	Neoadjuvant		257 (22.10%)	1 (16.67%)	256 (22.13%)	
Treatment with radiation therapy	No	4329 (100%)	2041 (47.15%)	15 (50.00%)	2026 (47.13%)	0.75
	Yes		2288 (52.85%)	15 (50.00%)	2273 (52.87%)	
Radiation therapy type	Adjuvant	1827 (42.2%)	1817 (99.45%)	10 (100.00%)	1807 (99.45%)	1.00
	Neoadjuvant		10 (0.55%)	0 (0.00%)	10 (0.55%)	
Treatment with immunotherapy	No	4329 (100%)	3836 (88.61%)	28 (93.33%)	3808 (88.58%)	0.57
	Yes		493 (11.39%)	2 (6.67%)	491 (11.42%)	
Immunotherapy type	Adjuvant	295 (6.8%)	222 (75.25%)	1 (100.00%)	221 (75.17%)	1.00
	Neoadjuvant		73 (24.75%)	0 (0.00%)	73 (24.83%)	
Treatment with hormone therapy	No	4329 (100%)	1653 (38.18%)	17 (56.67%)	1636 (38.06%)	**0.04**
	Yes		2676 (61.82%)	13 (43.33%)	2663 (61.94%)	
Hormone therapy type	Adjuvant	1935 (44.7%)	1835 (94.83%)	10 (100.00%)	1825 (94.81%)	1.00
	Neoadjuvant		100 (5.17%)	0 (0.00%)	100 (5.19%)	
Breast reconstruction	False	4329 (100%)	3908 (90.27%)	27 (90.00%)	3881 (90.28%)	1.00
	True		421 (9.73%)	3 (10.00%)	418 (9.72%)	

*Note*: All counts and percentages are summarized out of non‐missing entries. *p*‐values in bold are statistically significant at the *p*<0.05 level.

^a^ The No. (%) column represents the number of patients and percent out of total with non‐missing data for each variable.

To contextualize treatment outcomes among patients who were incarcerated, the EHR was subsequently reviewed by a member of the research team (YI). Patient charts were searched for common reasons cited in the literature for healthcare disparities among populations experiencing incarceration.^5^, ^8^ The following set of terms were searched in all patient charts: “delay/delayed,” “cancel/canceled,” “missed,” “no show,” “no response,” “adherence/nonadherence,” and “compliance/noncompliance.” Search results were extracted from the EHR, and frequencies of each unique mention of these key words were recorded for all patients with incarceration experience. The same process was applied to a random sample of patients selected from the nonincarcerated group as a comparison.

## 3 RESULTS

Of the 4329 patients with analyzable data, 30 (0.7%) were actively incarcerated at the time of diagnosis or DI while 4299 (99.3%) had no incarceration history (NI). Patient demographics are summarized in Table 1. Compared to patients who were not incarcerated, patients who were incarcerated at diagnosis/treatment were significantly younger at diagnosis (median age 60.4 years [NI] vs 49.4 years [DI], *p* < 0.001), more likely to be unmarried (18.2% [NI] vs 68.4% [DI], *p* < 0.001), more likely to have substance use documented at diagnosis (3.1% [NI] vs 20.0% [DI], *p* = 0.01), and less likely to have alcohol use documented at diagnosis (45.9% [NI] vs 14.3% [DI], *p* = 0.02). No differences in race/ethnicity, BMI, or tobacco use at diagnosis were observed between these groups.

Patients who were incarcerated at diagnosis/treatment were significantly more likely to have a family history of breast cancer compared to patients who were not incarcerated (41.5% (NI) vs 63.3% (DI), p = 0.02). They were also significantly less likely to have a family history of other cancers compared to patients who were not incarcerated (62.5% (NI) vs 43.4% (DI), p = 0.03). No differences in family history of BRCA mutation rates were observed between patients who were incarcerated at diagnosis/treatment and patients with no incarceration history. There were no statistically significant differences in tumor grade, estrogen receptor (ER) status, progestin receptor (PR) status, or HER2 status between patients who were incarcerated at diagnosis/treatment and patients with no incarceration history. Similarly, no differences in pathological stage, clinical stage, or summary stage were observed (Table 1).

Treatment patterns were similar between the two groups. Among patients undergoing surgery, rates were similar for lumpectomy (35.8% [NI] vs 38.5% [DI]), mastectomy (46.9% [NI] vs 38.5% [DI]), and no surgery (17.3% [NI] vs 23.1% [DI], *p* = 0.62). For treatment with chemotherapy, approximately 41.1% of patients with no incarceration history and 33.3% of patients who were incarcerated received chemotherapy (*p* = 0.39). Proportions of patients receiving neoadjuvant (22.1%, [NI] vs 16.7% [DI]) and adjuvant therapy (77.9% [NI] vs 83.3% [DI], *p* > 0.99) were similar; notably, only one patient who was incarcerated was documented to have received neoadjuvant chemotherapy. Radiation therapy followed these trends with 52.9% of patients with no incarceration history and 50.0% of patients who were incarcerated during diagnosis/treatment undergoing radiotherapy (*p* = 0.75). All patients who were incarcerated (100.0%) and most patients who were not incarcerated (99.5%) received adjuvant radiation therapy (*p* > 0.99). While not statistically significant, 11.4% of patients who were not incarcerated received immunotherapy and only 6.7% of patients who were incarcerated at diagnosis/treatment received immunotherapy (*p* = 0.57). Patients who were incarcerated were significantly less likely to receive endocrine therapy than patients who were not incarcerated (61.9% [NI] vs 43.3% [DI], *p* = 0.04). Breast reconstruction rates were similar among patients who were incarcerated vs not incarcerated (9.7% [NI] vs 10.0% [DI], *p* = 1.0).

**FIGURE 1:**
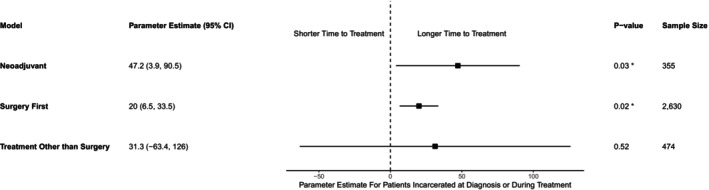
Forest plot of time from diagnosis to treatment for individuals who were incarcerated at diagnosis/treatment versus not incarcerated. *indicates statistical significance at the 0.05 level.

In the linear regression model, patients who were incarcerated had a longer time from diagnosis to initiation of neoadjuvant chemotherapy compared to patients who were not incarcerated (+47.2 days on average, 95% CI 3.9–90.5, *p* = 0.03; Figure 1). For patients undergoing surgery first, patients who were incarcerated at diagnosis/treatment also had an increased time from diagnosis to surgery compared to the not‐incarcerated group (+20 days on average, 95% CI 6.5–33.5, *p* = 0.02). For patients who did not undergo surgery, but did undergo at least one other type of treatment, there was no statistically significant difference in time from diagnosis to treatment between the two groups (estimate 31.3, 95% CI‐63.4 to 126, *p* = 0.52).

**FIGURE 2:**
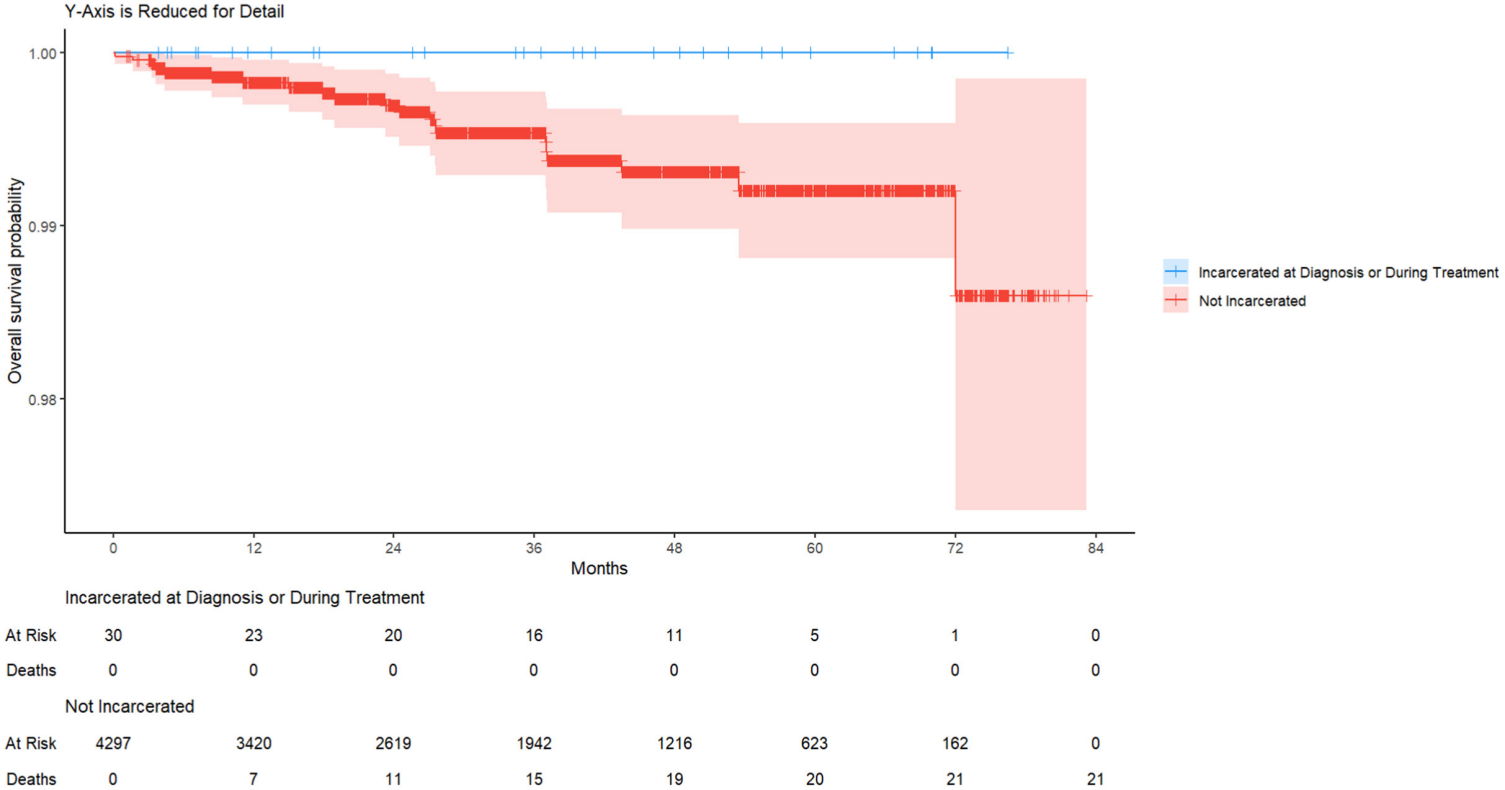
Unadjusted overall survival for individuals who were incarcerated at diagnosis/treatment versus not incarcerated (log‐rank *p* = 0.70).

There were no recorded deaths in the group of patients who were incarcerated at diagnosis/treatment during the study period. For patients who were not incarcerated, there were 21 (0.5%) recorded deaths. There was no statistically significant difference in OS between patients who were incarcerated at diagnosis/treatment and patients who were not incarcerated (12‐month OS rate [95% CI]: incarcerated at diagnosis/treatment 1 [1], not incarcerated 0.998 [0.996–0.999]; 60‐month OS rate [95% CI]: incarcerated at diagnosis/treatment 1 [1], never incarcerated 0.992 [0.987–0.995]; log‐rank *p* = 0.70; Figure 2).

### 3.1 Key word results

**FIGURE 3:**
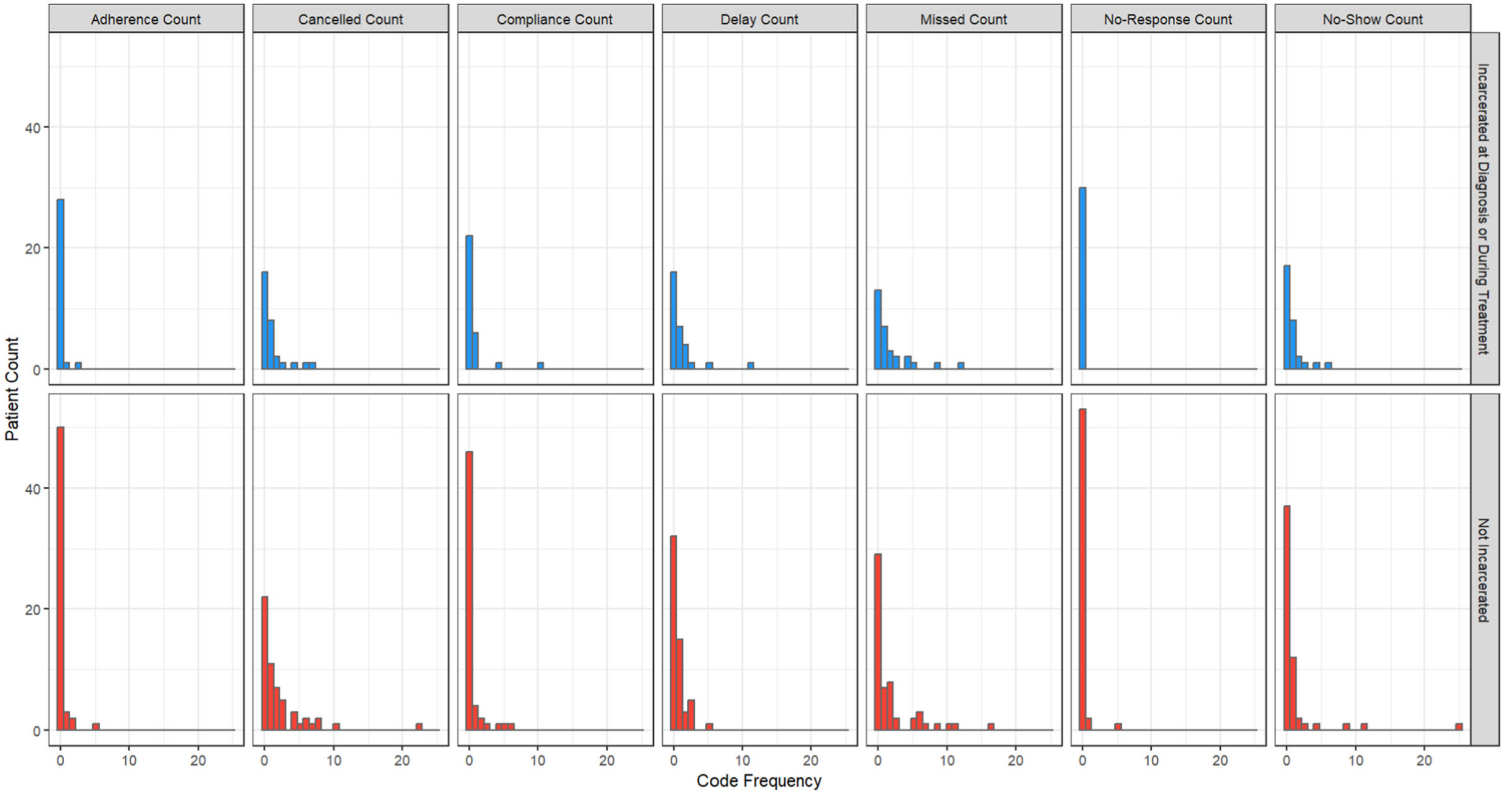
Bar graph of key term frequencies for individuals who were incarcerated at diagnosis/treatment versus not incarcerated.

The distribution of key words for patients who were incarcerated at diagnosis/treatment vs patients with no incarceration history are summarized in Figure 3. Patients who were incarcerated at diagnosis or DI numerically had more “missed” term counts, however, this difference was not statistically significant. Patients who were not incarcerated had a trend towards having more “canceled” counts, however, this did not reach statistical significance. Overall, there were no significant differences in key term frequencies between the two groups.

## 4 DISCUSSION

In this study of individuals who are incarcerated and received a breast cancer diagnosis and/or treatment at a tertiary care center, we appreciated significant differences in patient characteristics and treatment patterns compared to patients with no incarceration history. Patients who were incarcerated at diagnosis or DI were younger and more likely to have a family history of breast cancer. They also experienced significant treatment delays in the time of diagnosis to neoadjuvant chemotherapy and surgery despite having no differences in cancer stage at presentation. We believe ours is the first study to assess treatment patterns for breast cancer in an incarcerated population and been able to quantify the delays that are often anecdotally appreciated.

Greater time‐to‐surgery (TTS) has been associated with lower overall and disease‐specific survival in breast cancer.^23^ Socioeconomic factors are significant predictors for longer TTS and initiation of other cancer treatments, which has been more commonly appreciated among patients who are Black or Hispanic, older, on Medicaid or uninsured, in urban settings, and seeking treatment at high‐volume facilities.^24^, ^25^, ^26^ Incremental delays in initiation of cancer treatment, including surgery, have been associated with proportionally increased disease‐specific mortality.^27^, ^28^ While there are currently no prescribed guidelines for recommended number of days between diagnosis and first treatment, the improved associations between shorter times to treatment initiation suggest the potential value of incorporating more rigorous time‐specific metrics.^29^ Prior studies have suggested that ~8 weeks or 60 days as a TTS benchmark, after which worse survival is observed.^30^, ^31^ The incarcerated population in the US is disproportionately comprised of individuals from marginalized racial/ethnic and socioeconomic groups.^17^, ^32^ We posit that the longer time from diagnosis to initiation of neoadjuvant therapy (47 days on average) and time from diagnosis to surgery (20 days on average) reflect the compounded effects of preexisting sociodemographic disparities and the barriers to care coordination inherent to the carceral system and that further impede timely care, as have been shown in other cancers.^33^ Provision of metrics and guidelines for timely treatment initiation and diagnosis‐to‐treatment intervals may be particularly important for patients experiencing incarceration, who are more susceptible to delays in care.

Literature on cancer prevalence in populations interfacing with the criminal legal system is generally sparse. Puglisi et al's 2020 study of the National Survey on Drug Use and Health reported a similar prevalence of breast cancer among adults with and without criminal justice involvement and concluded the similar rates may reflect underdiagnosis due to lower screening practices in prisons. Pickett et al's 2018 study on two urban jails reported fewer than 40% of women prisoners had up‐to‐date mammograms.^14^ Screening metrics were outside of the scope of our study; however, lower mammography rates in carceral facilities may have led to an overall underdiagnosis of breast cancer and smaller study population.^7^ Additionally, lower screening rates may be partially attributable to the much smaller proportion of women in the carceral system compared to the proportion of women in the general population.^34^

Breast cancer‐associated mortality outcomes across populations experiencing incarceration are not well described. According to the Department of Justice report from 2001 to 2019, cancer mortality rates were higher in state prisons compared to the general US population, adjusting for sex, race or ethnicity, and age distribution (state prison mortality rate 88/100,000; US resident mortality rate 83/100,000).^2^ However, prison mortality reports by federal agencies do not differentiate cancer types; thus, breast cancer specific mortality is difficult to discern.^2^ A 2005 study by Mathew et al. compared cancer mortality among incarcerated populations at the University of Texas Medical Branch in Galveston, Texas, to a random Surveillance, Epidemiology, and End Results (SEER) sample over a 20‐year period.^35^ They reported on 49 patients with breast cancer and 15 (1.4%) deaths were appreciated. Our study had fewer deaths, which may be due to our study taking place roughly 30 years later, geographic differences, and patients being exclusively referred to the University of Texas Medical Branch in Mathew et al.'s study (whereas in our study, it is possible that some patients who required acute care and possibly died were evaluated at hospitals closer to the prison).^35^ The small sample and scarcity of contemporary reports on breast cancer mortality in US prisons continue to limit any ability to make meaningful comparisons.^8^ Robust data tracking the incidence, prevalence, and mortality outcomes in state and federal prisons across cancer types are necessary for understanding how specific cancers may be disproportionately impacting this population and how we may intervene to reduce disparities.

Clear contributors to the breast cancer treatment disparities we appreciated did not emerge from our qualitative examination. We had hypothesized that patients who were incarcerated would have significantly higher code frequencies for “canceled,” “no show,” or “missed” appointments, issues with treatment “adherence” or “compliance”, and “no response” due to challenges with communication between appointments. While these issues are likely still present at the individual patient level, they may not be as significant contributors compared to more location‐specific barriers to care coordination, such as lack of transportation from the prison to the hospital.^33^ Future studies should consider qualitative interviews with individuals who are incarcerated, prison healthcare personnel, and physicians at tertiary care centers to identify these barriers. More intensive EHR review to assess major contributors to delays in care may also be beneficial to follow this study.

### 4.1 Limitations

This study has several limitations. The sample size is small given the smaller population of women who are incarcerated compared to men.^34^ While the Department of Adult Corrections cites the tertiary care center from which this data was collected as the primary referral site, it is possible that some patients who are incarcerated across the state received their cancer care elsewhere. This study did not include individuals incarcerated in federal prisons, tribal systems, jails, or who were previously incarcerated in other states, which may limit the generalizability of the findings. Finally, data quality for individuals experiencing incarceration is generally poor due to lack of EHR interoperability and standardization across carceral settings.^36^, ^37^ To mitigate these risks, incarceration status was confirmed through direct EHR review and qualitative data were included; however, improving data quality requires structural change at all stages of data collection, storage, and accessibility. Future studies may benefit from collecting data directly from state and federal prisons, linking with specialty care data, and including multiple prisons to characterize broader trends in care.

## 5 CONCLUSIONS

Patients who are incarcerated at the time of breast cancer diagnosis or treatment experience delays in surgery and treatment initiation. Though no significant differences in mortality were appreciated, these findings are concerning for missed treatment opportunities within the carceral system. Further research is necessary to understand the full scope of disparities and the systemic factors that contribute to them to develop effective interventions.

## AUTHOR CONTRIBUTIONS

**Yoshiko Iwai:** Conceptualization (lead); data curation (equal); investigation (equal); methodology (equal); writing – original draft (lead); writing – review and editing (lead). **Alice Yunzi L. Yu:** Conceptualization (supporting); formal analysis (equal); investigation (supporting); writing – review and editing (equal). **Samantha M. Thomas:** Data curation (lead); formal analysis (lead); methodology (equal); software (equal); validation (lead); visualization (lead); writing – original draft (equal); writing – review and editing (equal). **Tyler Jones:** Data curation (equal); formal analysis (equal); investigation (equal); software (equal); validation (equal); writing – original draft (supporting); writing – review and editing (equal). **Kelly E. Westbrook:** Formal analysis (supporting); investigation (equal); writing – review and editing (equal). **Andrea K. Knittel:** Conceptualization (equal); formal analysis (equal); investigation (equal); methodology (equal); supervision (supporting); writing – original draft (equal); writing – review and editing (equal). **Oluwadamilola M. Fayanju:** Conceptualization (equal); data curation (equal); formal analysis (equal); investigation (equal); methodology (equal); resources (lead); supervision (lead); writing – original draft (equal); writing – review and editing (equal).

## FUNDING INFORMATION

Dr. Fayanju is supported by the National Institutes of Health (NIH) under Award Number 1K08CA241390 (PI: Fayanju) and P50CA244690 (PI: Bekelman), the Breast Cancer Research Foundation, and philanthropic funds from the Haas family. She also reports research support unrelated to this work from Gilead Sciences, Inc. This work was also supported by the NIH under Award Number 2P30CA016520–45 (PI: Vonderheide). SM Thomas is supported by the NIH under award numbers 5P30‐CA014236‐50 (PI: Kastan), 5UL1TR002553–05 (PI: Li), and 5R01DA047301–05 (PI: Vilardaga). SMT also reports foundational funding from the V Foundation and the Fullerton Foundation. The content of this manuscript is solely the responsibility of the authors and does not necessarily represent the official views of the NIH.

## CONFLICT OF INTEREST STATEMENT

None of the other authors has any relevant conflicts of interest to disclose.

## Data Availability

Data available on request due to privacy/ethical restrictions.
